# Being overindebted and overweight in Switzerland – A largely unexplored association in an understudied population

**DOI:** 10.1371/journal.pone.0342080

**Published:** 2026-02-17

**Authors:** Oliver Hämmig

**Affiliations:** Epidemiology, Biostatistics and Prevention Institute (EBPI), University of Zurich, Zurich, Switzerland; University of Modena and Reggio Emilia: Universita degli Studi di Modena e Reggio Emilia, ITALY

## Abstract

**Objectives:**

Research on overindebtedness in general and on the relationship between overindebtedness and being overweight or obese in particular is extremely rare or practically nonexistent although both phenomena have shown an increasing trend in recent years and are expected and found to be more prevalent among lower social classes and educational levels. However, no such study for Switzerland has ever been conducted until now.

**Methods:**

Survey data collected in 2019 from 219 overindebted adult clients of four official debt advisory centers in the Canton of Zurich were used and linked with a sample of 1,997 respondents of the Swiss Health Survey 2017 of the same age and canton of residence. The entire study population included a total of 2,216 adult individuals. Contingency tables with relative frequencies were calculated to study differences between the two subsamples. Furthermore, logistic and Poisson regression analyses were performed to calculate unadjusted and multiple-adjusted odds ratios and risk ratios as proxies and measures for the relative risk of being overweight or obese among overindebted people.

**Results:**

The prevalence rates of being overweight and having a body mass index (BMI) of 25+ and particularly of being obese (BMI of 30+) were significantly higher among overindebted individuals (BMI ≥ 25: 46%, BMI ≥ 30: 15%) than predominantly non-overindebted people (BMI ≥ 25: 38%, BMI ≥ 30: 9%). Overindebtedness increased the odds or the relative risk for such unfavourable body weights by 20% to 36% (overweight) and by 59% to 70% (obesity) depending on the effect measure considered. This was found regardless of overindebted individuals’ sex, age and educational level and independent of the fact that they have a comparably very low sense of control, feel lonely much more often and show much more often moderate to severe depressive symptoms.

**Conclusions:**

Measures of effect or association found were statistically significant at least for obesity, but smaller than expected and somewhat under- and simultaneously overestimated in view of the younger average age and the lower educational level of the overindebted individuals.

## Introduction

Overindebtedness has increased in Switzerland and elsewhere over the course of the last two decades [1]. It has been observed and described as a ‘hidden epidemic’ in rich OECD countries [2]. Furthermore, overindebtedness has been recognized as a major risk factor for mental health. However, it is still a largely neglected phenomenon in health research and is particularly poorly explored in Switzerland and most other European countries. Some studies from Scandinavian countries and Germany and most of the recent studies from Switzerland have focused predominantly on the association between overindebtedness and mental health [3–15]. However, there are very few studies on other possible health-related correlates or outcomes of overindebtedness [15–20] and/or from countries other than those mentioned [18,21,22]. Aside from those focusing on medication use [6,7,23], alcohol and/or tobacco consumption [17,18], physical (in)activity [18] and problem gambling [19,20], studies on the impact of overindebtedness on health-related behaviours are rare. In particular, with only one exception [24], there has been no study to date on overindebtedness and obesity, another important epidemic and major public health problem.

Although the phenomena of both overindebtedness and obesity have shown an upwards trend in recent years [1,25], are considered an epidemic [2,26] and are expected to be more prevalent in lower social classes and educational levels [11,27–31], they have not been studied as closely related to one another, except for one study in Germany [24].

Against this background, the present study explores a possible relationship between overindebtedness and obesity. This question is of particular interest and relevance because it has been unexplored for Switzerland so far and because the two phenomena are relatively common and growing and possibly interrelated. But in contrast to the mentioned German study, the present Swiss study not (only) considers depression or smoking as possible mediators (or confounders) [24]. Apart from depression the study particularly takes proven antecedents, predictors or correlates of such mental (ill-)health conditions and (poor) health behaviours into account, namely one’s sense of control and feelings of loneliness. These two beliefs or emotional states were previously found to be closely related to overindebtedness [8,14,18] and are expected to be associated with overweight and obesity [32–34]. Consequently they are assumed to potentially mediate (or confound) the studied relationship between overindebtedness and obesity. At the same time, they are likely to increase (sense of control) or decrease (feelings of loneliness) with age and social status or educational level. All these influencing factors and potentially mediating variables need to be considered when studying the relationship between overindebtedness and overweight in order to detect possible mediation (or confounding).

## Methods

### Data and study population

Overindebted households or individuals are permanently or temporarily unable to meet their financial obligations and commitments and to repay and/or service all debts and outstanding accounts fully and on time [1,8,9]. This applies to all the advice seeking adult clients of the official debt advisory centers in the Canton of Zurich, who are thus overindebted by definition and in 2019 were asked to participate in a written survey on the basis of many health-related questions identically used in the nationally representative Swiss Health Survey. Data were collected for research purposes only between January and August 2019. In total, 219 clients of the four official debt advisory centers in the Canton of Zurich completed and returned the questionnaire. This sample was then merged with a comparable and selected subsample of the Swiss Health Survey of 2017, namely 1,997 respondents from the Canton of Zurich aged 18 years and older. The 219 voluntarily and anonymously participating clients of the debt advisory centers were categorized into the “exposed” group, and the 1,997 respondents from the Swiss Health Survey were categorized into the “non-exposed” group. The two merged samples and data sets covered a total number of 2,216 adult individuals living in the Canton of Zurich.

Since delicate data on overindebtedness are usually not covered and collected and/or overindebted individuals most likely are massively underrepresented in population-based surveys (due to self-selection) and since empirical evidence on overindebted individuals was completely lacking for Switzerland, such a combination of own collected survey data and secondary survey data and a study population with both “cases” (overindebted individuals) and “controls” (representatives of the general population) was intended from the very beginning of the study.

### Ethics approval and consent to participate

The present study including survey data from clients of the debt advisory centers does not fall within the scope of the Human Research Act (HRA) and was granted exemption from requiring ethics approval (Kanton Zürich, Kantonale Ethikkommission. BASEC-Nr. Req-2019–00173). The study is observational and not clinical or experimental and did not involve drugs, medical records or human tissues. The study is based on pooled survey data (self-reports) collected from a full sample of clients of different debt advisory centers (primary data) and from a random selection of the general population (secondary data). They were collected on a voluntary and anonymous basis, and in particular not from patients (in hospitals), pensioners (in retirement homes) or prisoners etc. Therefore no approval was required by the ethics committee nor any authorisation by the commissioner for data protection by the national and cantonal laws nor were these recommended by the medical-ethical guidelines for scientific integrity of the Central Ethics Committee and the Swiss Academies of Sciences.

Consent of all study participants and survey respondents was given explicitly by verbally agreeing to participate in the survey or by answering the questions asked and/or implicitly by returning the completed written questionnaire.

### Measures

Overindebtedness as the *exposure variable* was accordingly measured by being either an overindebted client of a debt advisory center and participating in the corresponding survey of 2019 or belonging to the group of the (mostly) not overindebted participants and respondents of the Swiss Health Survey in 2017.

The body mass index (BMI), which was subsequently calculated from self-reported height and weight, was used to measure whether someone was overweight or obese as the *outcome variable*.

Sense of control, loneliness and depression as the intervening or *mediating variables* were measured identically in both the surveys and subsamples. *Loneliness* was assessed by a single-item measure and the self-reported frequency of feeling lonely. People were directly asked about their feelings of loneliness and could answer on a 4-point response scale ranging from ‘seldom/never’ (1) to ‘very often’ (4). The *sense of control*, defined as the belief in having life events and chances under one’s own control, was measured by a short version of the Pearlin Mastery Scale (PMS-4) [14,35]. The short scale is based on four Likert-scale items or statements concerning being unable to solve one’s own problems, being pushed around in life, having little control over things in life or feeling helpless in dealing with problems. The four 4-point items with response options ranging from 1 ‘fully agree’ to 4 ‘fully disagree’ were summed up to a total score from 4 to 16, with the lowest sum score or range indicating being fatalistically ruled and feeling helpless (low sense of control) and the highest sum score or range indicating having one’s own life under control (high sense of control). *Depression* was assessed by a 9-item subscale of the 59-item Patient Health Questionnaire (PHQ-9). The PHQ-9 is a short and established measure of depressive disorders and severities [36].

Sex, age and education as the usual *control variables* were accordingly assessed by assigning oneself to a gender (0 ‘female’, 1 ‘male’), age category (18–20, 21–30, 31–40, 41–50, 51–60, 61–70, 71–80, 80 + yrs.) and the highest educational level achieved thus far (from 0 ‘no compulsory education’ to 7 ‘university degree’).

### Statistical analyses

First, stratified frequency or contingency tables were calculated to examine expected group differences regarding all study variables between overindebted clients of debt advisory centers and representatives of the general population.

Second, prevalence rates of exposure, mediator and outcome variables (overindebtedness, degree of sense of control, frequency of feelings of loneliness, overweight and/or obesity) stratified by two presumed important control variables (age, education) were calculated and illustrated as bar charts.

Third, multiple adjusted and stepwise logistic and Poisson regression analyses were carried out between overindebtedness as the main predictor variable and overweight and obesity as the two outcome variables. Both logistic and Poisson regression analyses were performed to calculate odds ratios (ORs) on the one hand and risk ratios (RRs) on the other as measures of association. When outcomes are rare, ORs and RRs are close together and ORs are good proxies for the relative risk. But when outcomes are common, the two ratios differ substantially from each other and ORs are not equally good estimates of the relative risk anymore. Since one of the studied outcomes here is very common (overweight), ORs in this study tend to systematically overestimate the relative risk of overweight depending on overindebtedness. Therefore both measures were calculated and compared with one another in order to evaluate and mutually validate the two estimates. Both different types of regression analyses were performed first by stepwise adjusting for control variables and subsequently by additionally including the three potentially mediating variables (sense of control, loneliness, depression) in the model.

## Results

The relative frequencies shown in Table 1 reveal significant differences between the two subsamples of the study population. Overindebted and professional advice seeking individuals are overproportionally male (53%, i.e., 5% above average), young or middle-aged (76%, i.e., 22% above average) and poorly or only moderately educated (73%, i.e., 24% above average) than predominantly non-overindebted representatives of the general population. Furthermore, compared with the general population, overindebted people have much more frequently no or a low sense of control (76% vs. 22%) and feel lonely much more often (43% vs. 4%). In addition, overindebted individuals show strongly increased prevalence rates of a moderate (30%) or severe (24%) depression compared with the general population (5% and 2%).

**Table 1:** Demographic and health-related characteristics of the study population (N = 2,216), stratified by subsamples.

		Clients of debt advisory centers (N = 219)^a^	Representatives of the general population (N = 1,997)^b^	Total study population (N = 2,216)
**Sex**	Female	47.2%	52.6%	**52.1%**
	Male	52.8%	47.4%	**47.9%**
**Age**	18-30 yrs. (young)	20.8%	14.3%	**15.0%**
	31-50 yrs. (middle-aged)	55.6%	36.9%	**38.7%**
	51-70 yrs. (elderly)	20.8%	32.3%	**31.2%**
	71 + yrs. (old)	2.8%	16.4%	**15.1%**
**Education**	Poor (0–1)	16.2%	10.0%	**10.6%**
	Intermediate (2)	56.5%	36.5%	**38.4%**
	Higher (3–5)	15.7%	20.2%	**19.8%**
	Highest (6–7)	11.6%	33.3%	**31.2%**
**Sense of control** (Pearlin Mastery Scale; PMS-4)	No control (4–8)	42.6%	4.7%	**8.5%**
	Low control (9–11)	33.3%	16.8%	**18.4%**
	Moderate control (12–14)	16.2%	39.3%	**37.0%**
	High control (15–16)	7.9%	39.2%	**36.1%**
**Loneliness**	Seldom/never feeling lonely	23.9%	63.7%	**59.8%**
	Sometimes feeling lonely	33.5%	32.8%	**32.9%**
	Fairly often feeling lonely	25.2%	2.0%	**4.3%**
	Very often feeling lonely	17.4%	1.5%	**3.1%**
**Depression** (Patient Health Questionnaire; PHQ-9)	No depression (0–4)	19.4%	67.8%	**63.0%**
	Mild depression (5–9)	26.7%	25.1%	**25.2%**
	Moderate depression (10–14)	30.0%	4.8%	**7.3%**
	Severe depression (15–27)	24.0%	2.3%	**4.5%**
**Body weight** (Body Mass Index)	Underweight (BMI < 18.5)	0.9%	3.1%	**2.9%**
	Normal weight (BMI = 18.5–25)	53.3%	58.9%	**58.4%**
	Slight overweight (BMI = 25–30)	31.1%	28.8%	**29.0%**
	Obesity (BMI ≥ 30)	14.6%	9.2%	**9.7%**

^a^Overindebted adults seeking advice from one of the official debt advisory centers in the Canton of Zurich and participating in the overindebtedness study (and survey) of 2019.

^b^Subsample of the nationally representative Swiss Health Survey of 2017, restricted to adult respondents (aged 18 yrs. and older) from the Canton of Zurich; unweighted data.

However, above all, overindebted individuals are slightly more often overweight (31% vs. 29%) and clearly more often obese (15% vs. 9%) than their non-overindebted counterparts.

**Fig 1:**
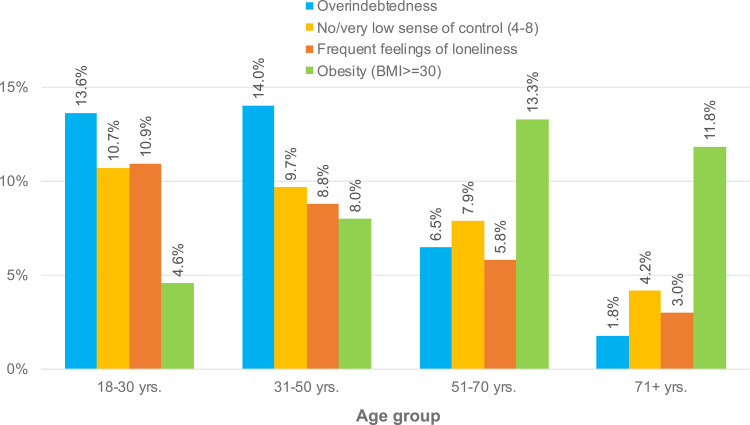
Prevalence of overindebtedness, no or very low sense of control, (very) frequent feelings of loneliness, and obesity in the study population by age group.

**Fig 2:**
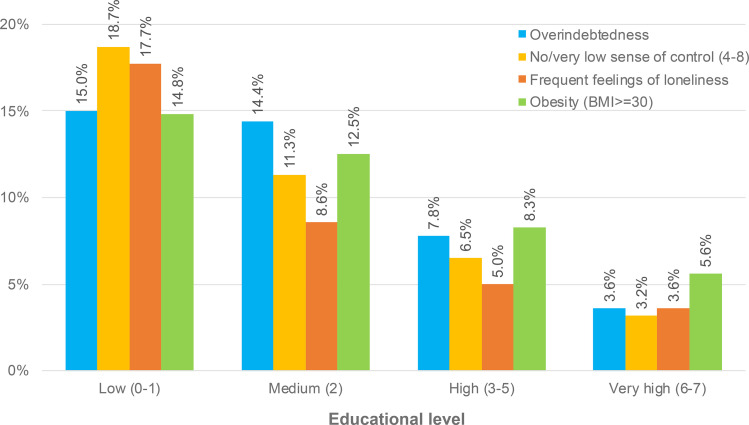
Prevalence of overindebtedness, no or very low sense of control, (very) frequent feelings of loneliness, and obesity in the study population by educational level.

Admittedly, this difference between the two groups under study regarding the prevalence of overweight and obesity is comparably small and systematically underestimated because the age of the overindebted individuals is clearly below average (see Table 1). Considering that younger people – although more at risk of being overindebted – are much less often overweight and particularly obese than older people are (see Fig 1), one might have expected that comparably young overindebted individuals are not more frequently overweight or obese than ordinary people with a higher average age. At the same time the group difference is presumably systematically overestimated because overindebted individuals are substantially less educated, and less educated people are usually and demonstrably more likely to be overweight and obese (see Fig 2). In other words, the effects of age and education may cancel each other out.

In fact, stepwise logistic and Poisson regression analyses revealed that age-adjusted odds ratios (ORs) and risk ratios (RRs) are significantly increased (above 1) compared with unadjusted ORs and RRs as measures of effect or association between overindebtedness and overweight (see Table 2). Additionally, adjusting for education (and sex) as expected reduced the two ratios again (see Table 2), but further adjusting for potential mediators or intervening variables such as sense of control, loneliness or depression did not make a great difference anymore (see Table 2). Overall, and in the fully specified regression models, an increased chance or risk was found in overindebted individuals for being overweight (OR: + 36%, RR: + 20%) and obese (OR: + 70%, RR: + 59%) relative to not overindebted ones.

**Table 2:** Unadjusted and adjusted associations of overindebtedness with overweight and obesity in the study population (N = 2,216).

	Overweight (BMI ≥ 25) (n = 849)				Obesity (BMI ≥ 30) (n = 213)			
	OR	95% CI	RR	95% CI	OR	95% CI	RR	95% CI
**Overindebtedness** (unadjusted)	**1.38**	1.04-1.83	1.21	0.98-1.49	**1.69**	1.12-2.55	**1.59**	1.09-2.33
Adjusted for								
**Sex** (male)	1.33	1.00-1.78	1.18	0.95-1.45	**1.68**	1.11-2.53	**1.58**	1.08-2.31
**+ Age** (1–8)	**1.66**	1.24-2.24	**1.35**	1.08-1.67	**2.14**	1.40-3.27	**1.96**	1.32-2.90
**+ Education** (0–7)	**1.42**	1.05-1.93	1.23	0.99-1.53	**1.75**	1.13-2.71	**1.64**	1.10-2.44
Additionally adjusted for								
**Sense of control** (4–16)	**1.41**	1.01-1.97	1.22	0.96-1.55	1.60	0.99-2.59	1.51	0.97-2.35
**+ Loneliness** (1–4)	**1.45**	1.03-2.04	1.24	0.97-1.59	**1.70**	1.04-2.78	**1.59**	1.01-2.51
**+ Depression** (0–27)	1.36	0.96-1.92	1.20	0.94-1.54	**1.70**	1.03-2.81	**1.59**	1.01-2.53

Bold print odds ratios (ORs) and risk ratios (RRs) = statistically significant at a 5% or lower level (see confidence interval).

Not surprisingly, estimates or effect sizes were smaller for calculated RRs than for ORs. This applies particularly to overweight as the much more common outcome (38.7%) than obesity (9.7%). In the case of overweight (BMI ≥ 25) both calculated ratios (ORs and RRs) were not even statistically significant anymore after adjusting for control variables and mediators (or confounders). However, at least for obesity (BMI ≥ 30) as the less common but more severe health-related outcome, overindebtedness was found to have a substantial and statistically significant, although not too strong effect. The relative risk of obesity was only slightly overestimated by performing logistic regression analyses and using ORs as measures of effect or association.

## Discussion

This study sought to reveal an association between overindebtedness and being overweight or obese. This association is completely unexplored in Switzerland since overindebted individuals are very rarely researched in this country, where very few previous (own) studies have found and shown a strong association of overindebtedness with poor mental health outcomes but have not focused thus far on overweight or obesity as an additional possible poor health outcome.

Notably, this study revealed an assumed association between overindebtedness and overweight or rather obesity, irrespective of the sex, age or educational level of the study participants, as three important predictors of overweight and obesity. This association was rather weak but still found among the study participants even after adjustment for sense of control, loneliness and depression, three proven correlates or outcomes of overindebtedness. This is in contrast to the previous findings of a very strong association between overindebtedness and poor mental health outcomes and an additional and substantial mediation observed by the sense of control [14]. In other words, effects or risk measures did not change substantially when important control variables and possible mediators or confounders were considered and simultaneously adjusted for in multivariate statistical analyses. Although overindebted individuals differ very strongly from the general populations as regards their much lower sense of control or mastery and their much more frequent feelings of loneliness and symptoms of depression, this does not at all explain their (significantly) increased relative risk of overweight and particularly obesity.

However, study results have also shown that the relatively young age of overindebted individuals has lead to a certain underestimation of the association between overindebtedness and overweight/obesity whereas their comparatively low educational level has resulted to some extent in an overestimation of the true association. But the two effects have largely cancelled each other out.

Prevalence rates of being overweight (31% vs. 29%) and obese (15% vs. 9%) were found to be slightly or clearly increased among overindebted people compared with those in the general population. Multiple logistic regression analyses revealed that overindebtedness increased the odds or the risk for overweight by 36% and for obesity by 70%. This number is somewhat lower than that reported in the German study by Münster and colleagues [24], the only other study published to date on the association between overindebtedness and overweight or obesity. In the German study of 2009, the sex-, age- and education-adjusted ORs found were 1.65 (overweight) and 2.42 (obesity), whereas in the present Swiss study, the ORs, likewise adjusted for sex, age and education, were 1.42 for overweight and 1.75 for obesity. In the German study, the prevalence rates of overweight (BMI ≥ 25) and obesity (BMI ≥ 30) were 57% and 25%, respectively, among the overindebted population compared with 44% and 11%, respectively, in a representative general population [24]. In the present Swiss study, these rates were 46% and 15% in the overindebted study population, whereas they were 35% and 9% in a random sample of the general population. Prevalence rates of overweight and obesity seem to be higher in Germany than in German-speaking Switzerland (or at least in the Canton of Zurich), and the increase in these rates due to overindebtedness was also found to be greater in Germany (+13%/ + 14%) than in German-speaking Switzerland (+11%/ + 6%).

The differences in the prevalence rates of overweight and obesity and/or in the effect sizes between the German study of Münster and colleagues [24] and the present Swiss study can have various reasons. They may simply reflect differences in the sociodemographic and socioeconomic structure and composition of the two countries, general populations or study samples. They may also reflect differences in the cultural “acceptance” or tolerance towards and the social discrimination against obesity and overindebtedness. Reasons may also lie in the social security against such risks of financial hardship and overindebtedness.

Differences in the effect sizes between the two studies can also have a methodological reason and be attributed to an artefact. Additional regression analyses besides the logistic ones in the present study revealed that estimates or effect sizes were overestimated as expected when ORs as measures of effect or association were used, particularly for a common outcome like overweight. Additionally calculated RRs (as measures for the relative risk) come closer to the “true” values or effects of overindebtedness on overweight and obesity. Given the fact that prevalence rates of overweight and obesity in the aforementioned German Study [24] compared to the present Swiss study are significantly higher in both the exposed (overindebted individuals) and the non-exposed groups (general population), ORs in the German study most probably even more strongly overestimated relative risks than in the Swiss study. This suggests that the “true” associations in the two studies are closer together and the differences between the findings of the two studies in fact are smaller than the different ORs indicate.

### Limitations

The study has revealed for the first time in this strongly underexplored population in Switzerland that overindebted individuals are significantly more likely to be overweight or rather obese and that this finding is quite robust and unlikely to be mediated, caused or confounded by some other influential factors or important third variables. However, the present study is limited in different ways:

- On the basis of the cross-sectional survey data used, the study cannot answer the question of whether overweight and obesity are actually a direct result of overindebtedness or whether overweight and obesity with increased probability may lead to disease, unemployment, divorce or separation, a low salary or job status and so on and, subsequently, to overindebtedness. In other words, owing to the design of the study, causality in the found relationship or association cannot be tested, nor can the direction of possible causality.
- Athough it cannot be tested with cross-sectional data if overindebtedness is more a cause or a consequence of obesity there is some indication and evidence from the research literature for more than correlation and even more than simple and unidirectional causation in the studied relationship between overindebtedness and obesity. In other words: There is a high plausibility for reverse causality. Bakkeli and Drange [15], in their nationwide Norwegian register study, examined reversed causality and the impact of health on overindebtedness and reported that poor health leads to increased debt or payment problems (e.g., debt enforcement and debt settlement). It has further been found and reported by different (longitudinal) studies that obesity has negative effects on income and wages [37–40] and on unemployment, reemployment and unemployment duration [41–43]. Low income and unemployment in turn are proven and strong risk factors for financial hardship and overindebtedness.
- Recruiting overindebted individuals from debt advisory centers carries the risk or potential of a selection bias. Clients of debt advisory centers may systematically differ in their personal characteristics, health status or degree of financial burden from other overindebted individuals who do not seek professional and official debt advise. The reasons of some overindebted individuals not to get help from debt advisory centers or to self-exclude from the study or survey participation might be systematically related to their body weight or BMI. Strongly obese and overindebted individuals may be less likely to register in a debt advisory centre and/or to participate in a health-related survey. This might somewhat limit the generalisability of the findings and lead to an underestimation of the true association between overindebtedness and obesity. It also could explain the comparably low prevalence rate of obesity among the overindebted individuals in the present Swiss study compared to the German study of Münster and colleagues [24].
- Misclassification bias also might have occurred. Overindebted individuals are expected to be underrepresented in a population-based survey such as many other socially disadvantaged groups. Nonetheless, they must be represented in a random sample of the general population. Since overindebted people in the Swiss Health Survey cannot be identified and classified as overindebted due to missing questions and information regarding overindebtedness, misclassification is likely to have occurred. This could have systematically biased the study results. However, this kind of misclassification of exposure is nondifferential, as it does not depend on body weight status or body mass index. A nondifferential misclassification would have biased the results in the direction of a systematic underestimation of the true associations or group differences. This means that the differences in the prevalence rates and relative risks of overweight and obesity between overindebted and non-overindebted people in reality are probably somewhat greater than those reported in the present study. Such an underestimation is much less problematic than an overestimation that might produce artefacts or significant but basically irrelevant or nonexistent findings.
- In addition to a misclassification bias in the assessment of exposure (overindebtedness) there is a strong possibility of a reporting or misclassification bias regarding the outcome (obesity). Self-reported BMI is usually lower than measured BMI. The use of self-reported height and weight instead of anthropometric measurements to assess the BMI is likely to result in a systematic underestimation of the prevalence of obesity [44–47]. However, this tendency to underreport weight and overreport height is more likely to occur in moderately obese individuals than in severely obese [48]. Some obese individuals therefore are misclassified as being non-obese, and this is a non-random misclassification that could lead to an overestimation rather than to an underestimation of the association between overindebtedness and obesity [45,48].

## Conclusion

Although overindebted individuals were slightly or clearly more likely to be overweight and obese than non-overindebted people from the general population, the higher prevalence and relative risk of overweight and obesity were much less pronounced than the very poor mental health conditions previously reported for overindebted individuals [8,9]. One must admit that the comparably weak association found between overindebtedness and overweight or obesity is not a strong indication for a causal relationship.

Even though there is no prove or strong indication of a causal relationship this does not mean that overindebtedness is not causing or at least contributing to obesity rather than simply going along with an increased prevalence or risk of obesity. This means that preventing overindebtedness or at least reducing the burden of overindebtedness can potentially help prevent overweight and obesity. This is of particular importance and public health relevance since the two phenomena, obesity and overindebtedness, are both on the rise and considered epidemics.

Overindebtedness in Switzerland is growing and not a marginal phenomenon. Estimated 6.5% to 8% of the total population are unable to serve their debts or have at least three kinds of debts or two kinds of payments in arrears [9]. This is a quite substantial proportion of the population. In addition, there is practically no way out of private insolvency and overindebtedness. As one of very few countries in Europe Switzerland does not know an official and complete debt relief procedure for overindebted individuals. When addressing obesity as a major public health problem, public health policy in future and Switzerland should more focus on preventing or reducing financial burden and hardship than solely on promoting physical activity and healthy diet. Helping overindebted people to get out of debt and offering them better prospects in life by making a complete debt relief possible could be a promising beginning.

## Supporting information

S1 File Questionnaire (in German) of the survey among 219 clients of debt advisory centers. (PDF)

S2 File Raw data of the survey among 219 clients of debt advisory centers (as Excel-File). (XLSX)
