# Supplementary material for: Being overindebted and overweight in Switzerland – A largely unexplored association in an understudied population
Source: PLoS One. 2026 Feb 17;21(2):e0342080. doi: 10.1371/journal.pone.0342080 (PMC12912557; doi:10.1371/journal.pone.0342080)
Supplement: S1 File — (PDF) [file pone.0342080.s001.pdf]

FRAGEBOGEN ZU SCHULDEN & GESUNDHEIT

Liebe Teilnehmerin, lieber Teilnehmer!

Ich freue mich sehr, dass Sie an dieser Umfrage zum Thema Schulden und Gesundheit, im Rahmen meiner Masterarbeit für die Universität Zürich teilnehmen. Ihre Antworten sind wichtig für die Schuldenberatung, die Schuldenprävention und die Wissenschaft. Das Ausfüllen des Fragebogens ist freiwillig und wird ca. 15 - 20 Minuten in Anspruch nehmen. Es ist wichtig, dass Sie alle Fragen beantworten. Danke, dass Sie sich Zeit nehmen.

Ihre Angaben werden vertraulich behandelt und anonym ausgewertet.

Bei Fragen zur Studie können Sie sich an Joanna Herzig (joanna.herzig@zuerich.ch) wenden.

Zu Beginn ein paar Fragen zu Ihrem Haushalt und Ihrer Arbeitssituation.

**1. Wie viele Personen leben in Ihrem Haushalt** (inkl. Sie selber)**?**	_______________
**2. Was trifft am besten auf Ihre Haushaltssituation zu?**	☐ Alleinlebend<br>☐ Ehepaar/Partnerschaft mit Kindern<br>☐ Ehepaar/Partnerschaft ohne Kinder<br>☐ Alleinerziehend<br>☐ Wohngemeinschaft<br>☐ Bei den Eltern wohnend
**3. Wie sieht Ihre berufliche Situation aus?** (mehrere Antworten möglich)	☐ Vollzeit erwerbstätig (90-100%)<br>☐ Teilzeit erwerbstätig (unter 50%)<br>☐ Teilzeit erwerbstätig (50%-85%)<br>☐ Arbeitslos<br>☐ Auf Stellensuche (Nicht als arbeitslos eingeschrieben)<br>☐ In Ausbildung (Schüler/Student)<br>☐ Im Militär/Zivildienst<br>☐ Hausfrau / Hausmann<br>☐ Rentenempfänger<br>☐ IV-Bezüger<br>☐ Sozialhilfeempfänger<br>☐ Anderes: ____________________
**4. Sind Sie aus gesundheitlichen Gründen nicht erwerbstätig?**	☐ Ja<br>☐ Nein
**5. Arbeiten Sie aus gesundheitlichen Gründen nicht Vollzeit?**	☐ Ja<br>☐ Nein

Im nächsten Teil werden Ihnen Fragen zu Ihrer finanziellen Situation gestellt.

6. **Wie hoch ist Ihr persönliches monatliches Nettoeinkommen** (Lohn, Alimente, Sozialhilfe, usw.)	___________
7. **Kriegen Sie einen 13. Monatslohn oder/und einen Bonus?**	☐ Ja, Höhe: ___________<br>☐ Nein
8. **Wo hoch ist ungefähr die Summe <u>aller</u> Nettoeinkommen pro Monat in Ihrem Haushalt?** (Wohngemeinschaft ausgeschlossen)	☐ Weniger als 3000 CHF<br>☐ 3001 CHF bis 4500 CHF<br>☐ 4501 CHF bis 6000 CHF<br>☐ 6001 CHF bis 7500 CHF<br>☐ 7501 CHF bis 9000 CHF<br>☐ Mehr als 9000 CHF
9. **Seit wie vielen Jahren haben Sie Schulden?**	☐ Seit mehr als 15 Jahren<br>☐ Seit mehr als 10 Jahren<br>☐ Seit mehr als 5 Jahren<br>☐ Seit mehr als 2 Jahren<br>☐ Seit weniger als 2 Jahren
10. **Wie hoch sind Ihre Schulden (ohne Hypothekarschulden) ungefähr?**	☐ Unter 5'000 CHF<br>☐ 5001 CHF – 10'000 CHF<br>☐ 10'001 CHF – 20'000 CHF<br>☐ 20'001 CHF – 40'000 CHF<br>☐ 40'001 CHF – 60'000 CHF<br>☐ 60'001 CHF – 80'000 CHF<br>☐ 80'001 CHF – 100'000 CHF<br>☐ Über 100'000 CHF
11. **Haben Sie Geld, um Schulden abzuzahlen?**	☐ Ja, immer<br>☐ Ja, manchmal<br>☐ Nein

**12. Welche der folgenden Situationen trifft am ehesten auf Sie zu?**	☐ Ich werde meinen Schulden in den nächsten 12 Monaten zurückzahlen können.<br>☐ Ich werde meine Schulden in den nächsten 3 Jahren zurückzahlen können.<br>☐ Ich werde meine Schulden in den nächsten 5 Jahren zurückzahlen können.<br>☐ Ich gehe davon aus, meine Schulden nicht mehr vollständig zurückzahlen zu können.
**13. Was sind die Gründe für Ihre Verschuldung?**<br>(Mehrere Antworten möglich)	☐ Arbeitslosigkeit<br>☐ Krankheit/Unfall<br>☐ Überforderung mit den Finanzen<br>☐ Heirat<br>☐ Trennung/Scheidung<br>☐ Bewusste Verschuldung<br>☐ Geburt/Adoption von Kindern<br>☐ Scheitern als Selbständigerwerbende/r<br>☐ Kaufsucht<br>☐ Alkoholsucht<br>☐ Drogensucht<br>☐ Spielsucht<br>☐ Tiefer Lohn (Workingpoor)<br>☐ Hohe Fixkosten<br>☐ Auszug aus dem Elternhaus<br>☐ Pensionierung<br>☐ Unterstützung anderer Personen<br>☐ Schulden verursacht durch andere Person<br>☐ Andere Gründe

**14. Von welchen Geldeintreibungsformen sind Sie zurzeit betroffen?** (Mehrfachantworten möglich)	☐ Mahnungen<br>☐ Ratenzahlungen<br>☐ Zahlungsbefehle<br>☐ Einkommens - & Sachpfändung<br>☐ Verlustscheine<br>☐ Konkurs
**15. Von was für Schulden sind Sie betroffen?** (Mehrfachantworten möglich)	☐ Krankenkassenschulden<br>☐ Steuerschulden<br>☐ Kredit<br>☐ Kreditkartenschulden<br>☐ Leasing<br>☐ Alimente<br>☐ Bussen und Geldstrafen<br>☐ Gerichts- und Verfahrenskosten<br>☐ Genugtuung/Schadenersatz<br>☐ Private Darlehen (Schulden bei Verwandten/Freunden usw.)<br>☐ Andere Schulden
**16. In welcher Form bezahlen Sie die Krankenkassen-Prämien?**	☐ Bar am Postschalter<br>☐ Onlinebanking<br>☐ Lastschriftverwahren (LSV): wird direkt vom Konto abgezogen<br>☐ Andere Form
**17. Wie hoch ist Ihre persönliche Jahresfranchise bei der Krankenversicherung?**	☐ 300 CHF<br>☐ 500 CHF<br>☐ 1000 CHF<br>☐ 1500 CHF<br>☐ 2000 CHF<br>☐ 2500 CHF

**18. Kriegen Sie finanzielle Unterstützung (Geld) von Personen aus Ihrem privaten Umfeld?**	☐ Ja, regelmässig<br>☐ Ja, ab und zu<br>☐ Nein
**19. Gibt es Personen, die Sie bei finanziellen Verpflichtungen administrativ unterstützen?** (z.B. Übersicht Rechnungen, Steuererklärung ausfüllen etc.)	☐ Ja, mehrere Personen<br>☐ Ja, eine Person<br>☐ Nein
**20. Wünschen Sie sich mehr solche Unterstützung von Familie, Freunden und Bekannten?**	☐ Ja, sehr<br>☐ Ja, manchmal<br>☐ Nein

Die nächsten Fragen beziehen Sich auf Ihr Wohlbefinden und Ihren Gesundheitszustand.

**21. Wie ist Ihr Gesundheitszustand im Allgemeinen?**	☐ Sehr gut<br>☐ Gut<br>☐ Mittelmässig<br>☐ Schlecht<br>☐ Sehr schlecht
**22. Wie schätzen Sie Ihre Lebensqualität im Allgemeinen ein?**	☐ Sehr gut<br>☐ Gut<br>☐ Weder gut noch schlecht<br>☐ Schlecht<br>☐ Sehr schlecht
**23. Haben Sie das Gefühl, dass Ihr Gesundheitszustand wegen Ihren Schulden beeinträchtigt ist?**	☐ Nein, überhaupt nicht<br>☐ Ja, zum Teil<br>☐ Ja, stark
**24. Wie oft kommen Sie pro Woche in Ihrer Freizeit durch körperliche Betätigung zum Schwitzen?** (Bsp. Rennen/Velofahren)	Anzahl Tage pro Woche (0-7): ____________

25. Wie häufig treiben Sie Gymnastik, Fitness oder Sport?

- ☐ (Fast) täglich
- ☐ Mehrmals wöchentlich
- ☐ Etwa 1 Mal pro Woche
- ☐ Etwa 1-3 Mal pro Monat
- ☐ Seltener als 1 Mal pro Monat
- ☐ Nie

26. Haben Sie mindestens eine chronische Krankheit? (Eine Krankheit, die Sie schon 6 Monate andauert oder ca. noch mind. 6 Monate andauern wird)

- ☐ Ja, körperliche
- ☐ Ja, psychische
- ☐ Ja, körperliche und psychische
- ☐ Nein

27. Hatten Sie in den letzten 4 Wochen eine oder mehrere dieser Beschwerden?

	Gar nicht	Ein bisschen	stark
Rücken oder Kreuzschmerzen?	☐	☐	☐
Allgemeine Schwäche, Müdigkeit, Energielosigkeit?	☐	☐	☐
Einschlaf- oder Durchschlafstörungen?	☐	☐	☐
Kopfschmerzen, Druck im Kopf oder Gesichtsschmerzen?	☐	☐	☐
Schmerzen in den Schultern, im Nacken oder in den Armen?	☐	☐	☐

28. Wie oft haben Sie in den letzten 7 Tagen folgende Medikamente genommen?

	Täglich	Mehrmals	1 Mal	Seltener/ Nie
Schlafmittel?	☐	☐	☐	☐
Mittel zur Beruhigung?	☐	☐	☐	☐
Mittel gegen Depression? (Antidepressiva)	☐	☐	☐	☐
Schmerzmittel?	☐	☐	☐	☐

**29. Wie häufig haben sich diese Situationen in den letzten 12 Monaten ergeben?**	Sehr häufig	Häufig	Manchmal	Nie
Ich habe ein Medikament aufgrund meiner finanziellen Situation nicht gekauft.	☐	☐	☐	☐
Ich ging nicht zum Arzt, weil ich das Gefühl hatte, die Rechnung nicht bezahlen zu können.	☐	☐	☐	☐
Ich ging nicht zum Zahnarzt/zur Dentalhygiene, weil ich das Gefühl hatte, die Rechnung nicht bezahlen zu können.	☐	☐	☐	☐
Ich konnte aus finanziellen Gründen, nicht an sozialen Aktivitäten teilnehmen (z.B. Sportveranstaltung, Kinobesuch, Essen gehen).	☐	☐	☐	☐

**30. Wie oft haben Sie sich in den letzten vier Wochen…**	Immer	Meistens	Manchmal	Selten	Nie
voller Leben gefühlt?	☐	☐	☐	☐	☐
sehr nervös gefühlt?	☐	☐	☐	☐	☐
so niedergeschlagen oder verstimmt gefühlt, dass Sie nichts aufmuntern konnte?	☐	☐	☐	☐	☐
ruhig, ausgeglichen und gelassen gefühlt?	☐	☐	☐	☐	☐
voller Energie gefühlt?	☐	☐	☐	☐	☐
entmutigt und deprimiert gefühlt?	☐	☐	☐	☐	☐
erschöpft gefühlt?	☐	☐	☐	☐	☐
glücklich gefühlt?	☐	☐	☐	☐	☐
müde gefühlt?	☐	☐	☐	☐	☐

**31. Wie häufig kommt es vor, dass Sie sich einsam fühlen?**	☐ Sehr häufig<br>☐ Ziemlich häufig<br>☐ Manchmal<br>☐ Selten/Nie

32. Wenn Sie über Ihr Leben nachdenken, inwiefern stimmen Sie folgenden Aussagen zu?

	Voll und ganz	Eher	Eher nicht	Überhaupt nicht
Ich werde mit meinen eigenen Problemen nicht fertig.	☐	☐	☐	☐
Ich fühle mich im Leben gelegentlich hin und her geworfen.	☐	☐	☐	☐
Ich habe wenig Kontrolle über Dinge, die ich erlebe.	☐	☐	☐	☐
Oft fühle ich mich meinen Problemen ausgeliefert.	☐	☐	☐	☐

33. Wie oft haben Sie sich im Verlauf der letzten 2 Wochen durch die folgenden Beschwerden beeinträchtigt gefühlt?

	Überhaupt nicht	An einzelnen Tagen	An mehr als der Hälfte der Tage	Beinahe Jeden Tag
Wenig Freude oder Interesse an Ihren Tätigkeiten	☐	☐	☐	☐
Niedergeschlagenheit, Schwermut oder Hoffnungslosigkeit	☐	☐	☐	☐
Schwierigkeiten ein- oder durchzuschlafen, oder vermehrter Schlaf	☐	☐	☐	☐
Müdigkeit oder Gefühl keine Energie zu haben	☐	☐	☐	☐
Verminderter Appetit oder übermässiges Bedürfnis zu essen	☐	☐	☐	☐
Schlechte Meinung von sich selbst; Gefühl, ein Versager zu sein oder die Familie enttäuscht zu haben?	☐	☐	☐	☐

	Überhaupt nicht	An einzelnen Tagen	An mehr als der Hälfte der Tage	Beinahe Jeden Tag
Schwierigkeiten, sich auf etwas zu konzentrieren, z.B. Zeitungslesen, Fernsehen	☐	☐	☐	☐
Waren Ihre Bewegungen oder ihre Sprache so verlangsamt, dass es auch andern auffallen würde? Oder waren Sie im Gegenteil «zappelig» oder ruhelos und hatten dadurch einen stärkeren Bewegungsdrang als sonst?	☐	☐	☐	☐
Gedanken, dass Sie lieber tot wären oder sich Leid zufügen möchten.	☐	☐	☐	☐

**34. Wie viele Menschen stehen Ihnen so nahe, dass Sie auf ihre Unterstützung zählen können, wenn Sie ein ernstes Problem haben?**	☐ Keine<br>☐ 1-2<br>☐ 3-5<br>☐ Mehr als 5
**35. Wie viel Interesse und Anteilnahme zeigen andere Menschen an dem, was Sie machen?**	☐ Sehr viel<br>☐ Viel<br>☐ Weder viel noch wenig<br>☐ Wenig<br>☐ Kein
**36. Wie einfach wäre es für Sie, Hilfe von Nachbarn zu bekommen, wenn Sie diese benötigen?**	☐ Sehr leicht<br>☐ Leicht<br>☐ Möglich<br>☐ Schwierig<br>☐ Sehr schwierig
**37. Gibt es Personen, mit denen Sie wirklich jederzeit über ganz persönliche Probleme sprechen können?**	☐ Ja, mehrere Personen<br>☐ Ja, eine Person<br>☐ Nein

**38. Vermissen Sie manchmal eine Person, mit der Sie jederzeit über ganz persönliche Probleme sprechen können?**	☐ Ja<br>☐ Nein
**39. Wie häufig unternehmen Sie etwas mit Freunden, Familie, Bekannten usw.?**	☐ Fast täglich<br>☐ Etwa 1 Mal pro Woche<br>☐ Etwa 1-3 Mal pro Monat<br>☐ Ein paar Mal pro Jahr<br>☐ Seltener<br>☐ Nie
**40. Woher erfahren Sie Unterstützung?** (Mehrfachantworten möglich)	☐ Partnerin / Partner<br>☐ Familie / Verwandte<br>☐ Freunde<br>☐ Nachbarn<br>☐ Arbeitskollegen<br>☐ Arbeitgeber / Vorgesetzte<br>☐ Verein<br>☐ Kirche<br>☐ Anderes: ______________
**41. Wie oft haben Sie in den letzten 12 Monaten an Glückspielen teilgenommen?** (Lotto, Sportwetten, Casino etc.)	☐ 2 Mal oder mehr pro Woche<br>☐ 1-7 Mal pro Monat<br>☐ 6-11 Mal pro Jahr<br>☐ Weniger als 6 Mal pro Jahr<br>☐ Nie
**42. Haben Sie in den letzten 12 Monaten Probleme aufgrund von Glücksspielen gehabt?**	☐ Nein<br>☐ Ja, Beziehungsprobleme (Streit, Trennung, Scheidung usw.)<br>☐ Ja, Geldprobleme (Schulden usw.)<br>☐ Ja, psychische Belastung (Stress, Schlafprobleme usw.)

43. Waren Sie wegen Spielproblemen in den letzten 12 Monaten in Behandlung?

☐ Ja

☐ Nein

44. Rauchen Sie, wenn auch nur selten?

☐ Ja

☐ Nein

45. Rauchen Sie täglich?

☐ Ja, Anzahl Zigaretten: ___________

☐ Nein

46. Wie häufig trinken Sie normalerweise alkoholische Getränke?

☐ 3 Mal oder mehr pro Tag

☐ 2 Mal pro Tag

☐ 1 Mal pro Tag

☐ Mehrmals pro Woche

☐ 1-2 Mal wöchentlich

☐ 1-3 Mal monatlich

☐ Seltener als monatlich

☐ Nie, abstinent

47. Wie häufig in den letzten 12 Monaten:

	Nie	Weniger als 1x im Monat	Jeden Monat	Jede Woche	(fast) täglich
Haben Sie 8 Gläser (für Männer) bzw. 6 Gläser (für Frauen) eines alkoholischen Getränks <u>auf einmal</u> getrunken?	☐	☐	☐	☐	☐
Haben Sie den Eindruck gehabt, nicht aufhören zu können mit Trinken, wenn Sie einmal damit angefangen haben?	☐	☐	☐	☐	☐
Sind Sie wegen dem Trinken nicht fähig gewesen das zu machen, was normalerweise von Ihnen erwartet wird?	☐	☐	☐	☐	☐
Haben Sie am Morgen Alkohol getrunken, um wieder in Schwung zu kommen?	☐	☐	☐	☐	☐

[illegible]

	Nie	Weniger als 1x im Monat	Jeden Monat	Jede Woche	(fast) täglich
Konnten Sie sich nicht mehr an das erinnern, was in der Nacht vorher passiert ist?	☐	☐	☐	☐	☐
Haben Sie Schuldgefühle, Gewissensbisse gehabt wegen ihrem Alkoholkonsum?	☐	☐	☐	☐	☐

Zum Schluss noch ein paar Fragen zu Ihrer Person. Bitte kreuzen Sie jeweils nur eine Antwort an.

**48. Welches Geschlecht haben Sie?**	☐ weiblich ☐ männlich
**49. Wie gross sind Sie?**	__________ (in cm)
**50. Wie schwer sind Sie?**	__________ (in kg)
**51. Wie alt sind Sie?**	☐ 18 – 20<br>☐ 21 – 30<br>☐ 31 – 40<br>☐ 41 – 50<br>☐ 51 – 60<br>☐ 61 – 70<br>☐ 71 – 80<br>☐ Über 80
**52. Was ist Ihr Zivilstand?**	☐ Ledig<br>☐ Verheiratet<br>☐ Verwitwet<br>☐ Geschieden<br>☐ Getrennt<br>☐ Eingetragene Partnerschaft<br>☐ Aufgelöste eingetrag. Partnerschaft

**53. Wie viele Kinder haben Sie?**	_____________
**54. Wie viele davon Leben noch in Ihrem Haushalt?**	_____________
**55. Wie viele Kinder insgesamt unterstützten Sie finanziell?**	_____________
**56. Wie viele weitere Personen werden von Ihnen finanziell unterstützt?**	_____________
**57. Was ist Ihr höchster Bildungsabschluss?**	☐ Sekundarschule<br>☐ Berufslehre<br>☐ Berufsmaturität<br>☐ Gymnasiale Maturität<br>☐ Höhere Fachschule (HF)<br>☐ Fachhochschule (FH) / Pädagogische Hochschule (PH)<br>☐ Universität / ETH
**58. Was für eine Staatsbürgerschaft haben Sie?**	☐ Schweizer/in<br>☐ Ausländer/in<br>☐ Doppelbürger/in (Schweizer Nationalität UND ausländische Nationalität)

Vielen herzlichen Dank für das Ausfüllen des Fragebogens!

Falls Sie noch etwas sagen/hinzufügen wollen können Sie dies gerne hier tun:
