# Supplementary material for: Being overindebted and overweight in Switzerland – A largely unexplored association in an understudied population
Source: PLoS One. 2026 Feb 17;21(2):e0342080. doi: 10.1371/journal.pone.0342080 (PMC12912557; doi:10.1371/journal.pone.0342080)
Supplement: Attachment — Submitted filename: renamed_ae1c6.pdf [file pone.0342080.s004.pdf]

Zurich, 6. November 2025

Dear Editor,

The manuscript entitled

Being overindebted and overweight in Switzerland – A largely unexplored association in an understudied population
(PONE-D-25-13328)

has now been revised.

Please find attached the revised manuscript along with my replies to the comments and points raised by the reviewers. All corrections and additions are highlighted in yellow. The deleted words, sentences or paragraphs are not marked.

Reviewer' comments and authors' replies

Reviewer #1:

Statistical analysis

- "However, the interpretation of ORs as proxies for relative risk warrants caution, especially given the prevalence of outcomes (~30-46%). When outcomes are common, ORs tend to overestimate the relative risk, which the authors do not explicitly address. Alternative measures such as prevalence ratios or risk ratios (via Poisson regression with robust error variance) could provide more interpretable estimates."
- Recommendation:
  "Methodological Clarity: Clarify the rationale for using ORs as proxies for relative risk, especially given the high prevalence of outcomes, and consider alternative models (e.g., Poisson regression) for more interpretable estimates."

I agree with the reviewer that calculating odds ratios as a proxy for the relative risk of being overweight among overindebted individuals is somewhat limited as overweight is not a rare phenomenon in Switzerland as elsewhere. Odds ratios are good measures for the relative risk when outcomes are rare, but less so when they are common. However, since the only existing and comparable study from another country (Germany) has carried out the same logistic regression analyses (see Discussion section), I would dislike giving it up completely. Nevertheless, I have additionally performed Poisson regression analyses and calculated risk ratios (RRs) as measures of association. The risk ratios as newly and additionally presented estimates in Table 2 show slightly smaller effect sizes than odds ratios. Not surprisingly, this applies mainly for overweight as the much more common outcome.
In the Methods, the Results and the Discussion section different new paragraphs have been formulated and inserted concerning this matter.

Results

- “However, the interpretation could benefit from a more nuanced discussion of the potential reverse causality—whether obesity could contribute to financial hardship—and the implications thereof. The authors briefly mention this but could elaborate further, considering the bidirectional nature of social and health determinants.”

- Recommendation:
  “Address Reverse Causality: Expand discussion on the possibility that obesity may influence financial status, and suggest longitudinal research to disentangle causality.”

This has now been more extensively addressed and elaborated in the Discussion section. See a new paragraph with several new references under “Limitations”.

Discussion / Limitations

- “The comparison with German data provides valuable context, but the authors should clarify that differences in prevalence rates and effect sizes may also reflect cultural, healthcare, and socioeconomic differences beyond mere sample characteristics.”

A paragraph on that has been inserted in the Discussion section.

- “Data Quality: Discuss the potential impact of self-reported BMI bias more explicitly, possibly referencing validation studies.”

Done. See a new paragraph on that at the end of the “Limitations” subsection in the Discussion section.

- “Sample Representativeness: Elaborate on the potential selection bias introduced by recruiting overindebted individuals from advice centres and how this might influence generalisability.”

Done (see new paragraph under “Limitations” in the Discussion section).

- “Statistical Power: Acknowledge the limited sample size of overindebted individuals and its implications for the robustness of findings.”

Considering the estimated prevalence of the phenomenon of overindebtedness in Switzerland between 6.5% and 8% (see Hämmig & Herzig 2022) the proportion of overindebted individuals of 10% in the study sample of 2,216 is not too low. The absolute number of 219 overindebted individuals also does not really limit the statistical power or the robustness of the findings. The proportion of overindebted individuals and the findings in the study are comparable with those of the only existing German study on this topic which has a much larger sample size (Münster et al. 2009).

- “Further Analyses: Consider stratified analyses by age and education to explore effect modification.”

The sample size – although not too small – is not large enough to do education- or age-stratified analyses. However, I do not see a theoretical foundation for an effect modification anyway or rather a biological reason for different associations in different age groups or educational levels. And just doing stratified analyses as an explorative approach would be inappropriate anyway in my view.

- “Visuals and Data Presentation: Ensure that figures and tables are clearly labelled and accessible, and consider including confidence intervals directly in figures for clarity.”

Due to small numbers in frequency distributions by age group and educational level confidence intervals are getting partly very large and mostly overlap. Anyway, bar charts in Figures 1 and 2 are not shown to demonstrate that age groups or educational levels differ significantly from one another in this regard but rather to illustrate patterns of distribution and to justify that all relevant study variables (exposure, outcome, mediators/confounders) and studied/considered phenomena (overindebtedness, low sense of control, feelings of loneliness, obesity) go along with each other.

- “Discussion Depth: Further explore potential mediators and confounders, and discuss policy implications more explicitly.”

Done. See different highlighted sentences in the Discussion and the Conclusions section.

Reviewer #2:

- “It would be good if you could provide an answer to why your research question is interesting and relevant already in the introduction.”

Done. See the yellow marked paragraph in the Introduction section.

- “Relatedly, while the literature on overindebtedness and obesity may be small there is a large literature on poverty and obesity. I would assume that poor people are more often overindebted (even if their debt levels are probably lower than for wealthier people). It seems to me that the association between overindebtedness and obesity should be more relevant if there is an association on top and above that with poverty, in the same way as it is more relevant if there is an association on top and above that with education (which you do show). If not, overindebtedness could be seen as just an alternative measure of poverty.
  Ideally you have alternative measures of income or poverty in your data, and you can explore whether a statistically significant association remains when poverty/income is accounted for. If you do not have comparable measures in both data sources, you could still compare how well overindebtedness predict overweight/obesity compared to how well poverty predicts it in the Swiss Health Survey. You can also compare your estimated association with comparable

associations between poverty and overweight/obesity in the literature for Switzerland in particular."

I do not agree. Overindebtedness and poverty in my view are fairly different phenomena, and data used do not include established and common measures of poverty anyway. Overindebtedness is currently increasing in high-income countries and is observed even among better earning individuals and households. Poverty in contrast is excluded by definition among higher earners. Therefore I do not want to make a connection or mixing between the two phenomena and research fields and traditions in the paper.
In my view and in the case and context of Switzerland it's more a low social or socioeconomic status measured by education and/or income that is associated with overindebtedness and obesity. Income is not available as an identical and comparable measure in both data sets. And education has been considered in the present study.

- "It would be good to mention other potential confounders or mediators suggested by the literature, that you cannot investigate. This will clarify the contribution of the paper in relation to the existing literature. Potential confounders or mediators that you cannot include in your study can also be mentioned as a limitation of the study."

Since the literature on overindebtedness and obesity is practically inexistent there are no (other) potential confounders or mediators suggested by the literature. Risk or influencing factors of overweight or obesity named in the literature include sex, age, education, income, depression and smoking. I have studied a few (sex, age, education) and considered another two (sense of control, feelings of loneliness) which are not mentioned in the scarce literature but which are found to be related with depression and smoking.
Nevertheless, I have now additionally considered and included depression as another possible mediator or confounder in the regression analyses. Depressive symptoms were assessed in the two merged surveys and data sets. I have not considered depression as another mediator before since it turned out to be strongly associated with one's sense of control and feelings of loneliness. Apart from depression I don't see the necessity of introducing or discussing other potential mediators or confounders which I cannot study or for which I don't have a rationale.

- "Sense of control can probably be a confounder just as well as a mediator. It is easy to imagine that a lower sense of control increases both the risk of becoming indebted and of becoming obese. It is also easy to imagine that having become indebted or obese lowers sense of control though. The study does not allow the investigation of directions of causality, but you could be more agnostic about it."

I do now consequently talk or write about possible/potential mediators <u>or confounders</u> in connection with the sense of control or feelings of loneliness..

- "It would be good to have more details on your included confounding and mediating variables. It is not clear exactly what variables are used in your regressions presented in Table 2. What does for example Age (1-8) mean? That you include eight different age categories in the regressions? Which categories? Or do you use the categories in Table 1 (only three categories). Why do you use the particular categories that you do? This comment applies also to education, sense of control, and feelings of loneliness."

For anonymity reasons in the overindebtedness survey age wasn't asked exactly but rather by age category. In Table 1 these eight categories were reduced to four age groups. The sum score of the Pearlin Mastery Scale ranging from 4 to 16 as used in regression analyses (see Table 2) has been categorized into four degrees of sense of control (from "no" to "high") in Table 1. And the seven assessed educational levels (see Table 2) were grouped into four categories (see Table 1). See original descriptions and additional clarifications (yellow marked) in "Measures".

- "What was the participation rate in the debt advisory center survey? That is, how many participants asked to participate did not do so?"

Out of 369 adult clients showing up in the observation period, 219 with sufficient language skills were preselected by the debt advisors. All of the preselected clients who were eligible, basically willing and finally asked to participate in the survey did so (see also Hämmig & Herzig 2022).

- "Do you have any idea about the likely number of overindebted individuals in the Swiss Health Survey? If the Swiss Health Survey is representative, it should probably be close to population levels? Is there some information in the survey that can be used to proxy the number? Having some idea would be useful to say something about the likely extent of misclassification. Is it likely to be minimal or sizeable?"

No, there is absolutely no information that could be used to proxy the number of overindebted individuals in the Swiss Health Survey. We just know from (other) population surveys and socially disadvantaged groups that marginalized and stigmatized people like unemployed, working poor or overindebted individuals usually are strongly underrepresented in population-based surveys. Therefore the extent of misclassification is presumably minimal rather than sizeable.

- "You discuss possible misclassification of exposure – I think that you refer to the possibility of some overindebted people in the Swiss Health Survey here? Clariy this."

Yes, because there must be some overindebted people in a random sample of the general population which cannot be identified and classified as such, misclassification is likely to have occurred.

.

- "In your `strengths and weaknesses´ you emphasize that you do not make any causal claims, while you still seem to want to make such claims in your conclusions. You should be more coherent. I think that you should avoid statements that you cannot back with your analysis in the conclusions. You can discuss possible reasons behind the association, but any statement that you cannot back is speculative, which should be clear."

I have shortened and reformulated the according paragraph in the "Conclusion" subsection.

Some small comments:

i) In the introduction: the sentence that the lack of prior studies is "particularly applicable to Switzerland" seems a bit unnecessary if there is only one prior study, from Germany.

The according sentence is now deleted.

ii) The statement about both a systematic underestimation and overestimation is confusing. When you adjust for both education and age the problem disappears, no? It would be more interesting if you could discuss likely confounders that you could not include, and the likely bias on your estimated odds ratio because of that.

I don't understand this point. Systematically underestimated effects due to misclassification of exposure (overindebtedness) do not disappear by adjusting for age or education. They have to be considered and acknowledged.

iii) I suggest removing the word "finally" in "the study simply wanted to demonstrate and has finally…" . It suggests to the reader that this is something that the literature have long wanted to do, and now finally you have been able to. The word simply also seems a bit unnecessary.

I agree and have deleted the word.

iv) In general think about word choices and language to make it as correct and to the point as possible.

I tried to do so and to do better in this regard when revising the manuscript.

v) Will the data collected at the debt advisory centers be freely available, I see only information about the Swiss Health Survey in the Data Centre.

No, in contrast to the data of the Swiss Health Survey the data collected from the debt advisory centers are not freely available.

I am confident now that all required or suggested changes have been made appropriately and according to the reviewers' comments. I Am now looking very much forward to the publication of our manuscript in the PLOS ONE.

Kind regards also on behalf of my co-author,

Oliver Hämmig
