# Supplementary material for: Being overindebted and overweight in Switzerland – A largely unexplored association in an understudied population
Source: PLoS One. 2026 Feb 17;21(2):e0342080. doi: 10.1371/journal.pone.0342080 (PMC12912557; doi:10.1371/journal.pone.0342080)
Supplement: Attachment — Submitted filename: Referee report oveindebted and obese plos one.pdf [file pone.0342080.s003.pdf]

Referee report on “Being overindebted and overweight in Switzerland – A largely unexplored association in an understudied population”

The study investigates the association between being overindebted and overweight/obese in the kanton of Zürich in Switzerland. It adjusts for the most obvious confounders, age, education and gender. It also investigates two potential mediators, feelings of loneliness and sense of control.

1. It would be good if you could provide an answer to why your research question is interesting and relevant already in the introduction.

2. Relatedly, while the literature on overindebtedness and obesity may be small there is a large literature on poverty and obesity. I would assume that poor people are more often overindebted (even if their debt levels are probably lower than for wealthier people). It seems to me that the association between overindebtedness and obesity should be more relevant if there is an association on top and above that with poverty, in the same way as it is more relevant if there is an association on top and above that with education (which you do show). If not, overindebtedness could be seen as just an alternative measure of poverty.

Ideally you have alternative measures of income or poverty in your data, and you can explore whether a statistically significant association remains when poverty/income is accounted for.

If you do not have comparable measures in both data sources, you could still compare how well overindebtedness predict overweight/obesity compared to how well poverty predicts it in the Swiss Health Survey. You can also compare your estimated association with comparable associations between poverty and overweight/obesity in the literature for Switzerland in particular.

3. It would be good to mention other potential confounders or mediators suggested by the literature, that you cannot investigate. This will clarify the contribution of the paper in relation to the existing literature. Potential confounders or mediators that you cannot include in your study can also be mentioned as a limitation of the study.

4. Sense of control can probably be a confounder just as well as a mediator. It is easy to imagine that a lower sense of control increases both the risk of becoming indebted and of becoming obese. It is also easy to imagine that having become indebted or obese lowers sense of control though. The study does not allow the investigation of directions of causality, but you could be more agnostic about it.

5. It would be good to have more details on your included confounding and mediating variables. It is not clear exactly what variables are used in your regressions presented in Table 2. What does for example Age (1-8) mean? That you include eight different age categories in the regressions? Which categories? Or do you use the categories in Table 1

(only three categories). Why do you use the particular categories that you do? This comment applies also to education, sense of control, and feelings of loneliness.

6. What was the participation rate in the debt advisory center survey? That is, how many participants asked to participate did not do so?

7. Do you have any idea about the likely number of overindebted individuals in the Swiss Health Survey? If the Swiss Health Survey is representative, it should probably be close to population levels? Is there some information in the survey that can be used to proxy the number? Having some idea would be useful to say something about the likely extent of misclassification. Is it likely to be minimal or sizeable?

8. You discuss possible misclassification of exposure – I think that you refer to the possibility of some overindebted people in the Swiss Health Survey here? Clariy this.

9. In your ` strengths and weaknesses ´ you emphasize that you do not make any causal claims, while you still seem to want to make such claims in your conclusions. You should be more coherent. I think that you should avoid statements that you cannot back with your analysis in the conclusions. You can discuss possible reasons behind the association, but any statement that you cannot back is speculative, which should be clear.

Some small comments:

i) In the introduction: the sentence that the lack of prior studies is "particularly applicable to Switzerland" seems a bit unnecessary if there is only one prior study, from Germany.
ii) The statement about both a systematic underestimation and overestimation is confusing. When you adjust for both education and age the problem disappears, no? It would be more interesting if you could discuss likely confounders that you could not include, and the likely bias on your estimated odds ratio because of that.
iii) I suggest removing the word "finally" in "the study simply wanted to demonstrate and has finally…" . It suggests to the reader that this is something that the literature have long wanted to do, and now finally you have been able to. The word simply also seems a bit unnecessary.
iv) In general think about word choices and language to make it as correct and to the point as possible.
v) Will the data collected at the debt advisory centers be freely available, I see only information about the Swiss Health Survey in the Data Centre.
