# Supplementary material for: Being overindebted and overweight in Switzerland – A largely unexplored association in an understudied population
Source: PLoS One. 2026 Feb 17;21(2):e0342080. doi: 10.1371/journal.pone.0342080 (PMC12912557; doi:10.1371/journal.pone.0342080)
Supplement: Peer review history

# Peer review: Decision Letter 0

**DOI:** 10.1371/journal.pone.0342080.r001

**Authors:** Fabrizio Ferretti

15 Aug 2025

Dear Dr. Hämmig,

Thank you for submitting your manuscript to PLOS ONE. After careful consideration, we feel that it has merit but does not fully meet PLOS ONE’s publication criteria as it currently stands. Therefore, we invite you to submit a revised version of the manuscript that addresses the points raised during the review process.

Please submit your revised manuscript by Sep 29 2025 11:59PM. If you will need more time than this to complete your revisions, please reply to this message or contact the journal office at plosone@plos.org . When you're ready to submit your revision, log on to https://www.editorialmanager.com/pone/ and select the 'Submissions Needing Revision' folder to locate your manuscript file.

- A rebuttal letter that responds to each point raised by the academic editor and reviewer(s). You should upload this letter as a separate file labeled 'Response to Reviewers'.
- A marked-up copy of your manuscript that highlights changes made to the original version. You should upload this as a separate file labeled 'Revised Manuscript with Track Changes'.
- An unmarked version of your revised paper without tracked changes. You should upload this as a separate file labeled 'Manuscript'.

If you would like to make changes to your financial disclosure, please include your updated statement in your cover letter. Guidelines for resubmitting your figure files are available below the reviewer comments at the end of this letter.

If applicable, we recommend that you deposit your laboratory protocols in protocols.io to enhance the reproducibility of your results. Protocols.io assigns your protocol its own identifier (DOI) so that it can be cited independently in the future. For instructions see: https://journals.plos.org/plosone/s/submission-guidelines#loc-laboratory-protocols . Additionally, PLOS ONE offers an option for publishing peer-reviewed Lab Protocol articles, which describe protocols hosted on protocols.io. Read more information on sharing protocols at https://plos.org/protocols?utm_medium=editorial-email&utm_source=authorletters&utm_campaign=protocols .

We look forward to receiving your revised manuscript.

Kind regards,

Fabrizio Ferretti, Ph.D.

Academic Editor

PLOS ONE

Journal Requirements:

When submitting your revision, we need you to address these additional requirements.

1. Please ensure that your manuscript meets PLOS ONE's style requirements, including those for file naming. The PLOS ONE style templates can be found at

https://journals.plos.org/plosone/s/file?id=wjVg/PLOSOne_formatting_sample_main_body.pdf and

https://journals.plos.org/plosone/s/file?id=ba62/PLOSOne_formatting_sample_title_authors_affiliations.pdf

2. We note that you have indicated that there are restrictions to data sharing for this study. For studies involving human research participant data or other sensitive data, we encourage authors to share de-identified or anonymized data. However, when data cannot be publicly shared for ethical reasons, we allow authors to make their data sets available upon request. For information on unacceptable data access restrictions, please see http://journals.plos.org/plosone/s/data-availability#loc-unacceptable-data-access-restrictions.

Before we proceed with your manuscript, please address the following prompts:

a) If there are ethical or legal restrictions on sharing a de-identified data set, please explain them in detail (e.g., data contain potentially identifying or sensitive patient information, data are owned by a third-party organization, etc.) and who has imposed them (e.g., a Research Ethics Committee or Institutional Review Board, etc.). Please also provide contact information for a data access committee, ethics committee, or other institutional body to which data requests may be sent.

b) If there are no restrictions, please upload the minimal anonymized data set necessary to replicate your study findings to a stable, public repository and provide us with the relevant URLs, DOIs, or accession numbers. Please see http://www.bmj.com/content/340/bmj.c181.long for guidelines on how to de-identify and prepare clinical data for publication. For a list of recommended repositories, please see https://journals.plos.org/plosone/s/recommended-repositories. You also have the option of uploading the data as Supporting Information files, but we would recommend depositing data directly to a data repository if possible.

Please update your Data Availability statement in the submission form accordingly.

3. For studies involving third-party data, we encourage authors to share any data specific to their analyses that they can legally distribute. PLOS recognizes, however, that authors may be using third-party data they do not have the rights to share. When third-party data cannot be publicly shared, authors must provide all information necessary for interested researchers to apply to gain access to the data. (https://journals.plos.org/plosone/s/data-availability#loc-acceptable-data-access-restrictions)

For any third-party data that the authors cannot legally distribute, they should include the following information in their Data Availability Statement upon submission:

1) A description of the data set and the third-party source

2) If applicable, verification of permission to use the data set

3) Confirmation of whether the authors received any special privileges in accessing the data that other researchers would not have

4) All necessary contact information others would need to apply to gain access to the data

4. Please include a separate caption for each figure in your manuscript.

5. Please include captions for your Supporting Information files at the end of your manuscript, and update any in-text citations to match accordingly. Please see our Supporting Information guidelines for more information: http://journals.plos.org/plosone/s/supporting-information.

6. If the reviewer comments include a recommendation to cite specific previously published works, please review and evaluate these publications to determine whether they are relevant and should be cited. There is no requirement to cite these works unless the editor has indicated otherwise.

**Additional Editor Comments:**

Subject: Decision on Manuscript PONE-D-25-13328 – Major Revision Required

Dear Prof. Hämmig,

Thank you for submitting your manuscript entitled “Being overindebted and overweight in Switzerland – A largely unexplored association in an understudied population” to PLOS One. We have now received and carefully considered reports from two expert reviewers, and I have read the manuscript in detail myself.

While your research addresses an important question and makes a potentially valuable contribution to the field, both the reviewers and I agree that the manuscript, in its current form, is not yet ready for publication.

The reviewers have highlighted several issues that will need to be carefully addressed in a major revision.

You will find the full referee reports appended below. I urge you to respond to each comment carefully in your revised manuscript and to provide a detailed response letter outlining how you have addressed each point.

We are open to considering a revised version that thoroughly addresses the concerns raised. While we cannot guarantee acceptance of the revised version, we believe that with substantial improvement, your manuscript could be suitable for publication in PLOS One.

Should you choose to revise, we ask that you submit your revised manuscript and response letter within 30 days. If you require additional time, please let us know in advance.

Thank you again for considering PLOS One for your work. We look forward to receiving your revision.

Sincerely,

Fabrizio Ferretti

Editor, PLOS One

fabrizio.ferretti@unimore.it

[Note: HTML markup is below. Please do not edit.]

Reviewers' comments:

Reviewer's Responses to Questions

**Comments to the Author**

1. Is the manuscript technically sound, and do the data support the conclusions?

Reviewer #1: Partly

Reviewer #2: Partly

**********

2. Has the statistical analysis been performed appropriately and rigorously?

Reviewer #1: Yes

Reviewer #2: Yes

**********

3. Have the authors made all data underlying the findings in their manuscript fully available?

The PLOS Data policy

Reviewer #1: No

Reviewer #2: No

**********

4. Is the manuscript presented in an intelligible fashion and written in standard English?

Reviewer #1: Yes

Reviewer #2: Yes

**********

Reviewer #1: PEER REVIEW REPORT

Manuscript Title: "Being overindebted and overweight in Switzerland – A largely unexplored association in an understudied population"

Reviewer: [Anonymous]

Date: 06 July 2025

1. Executive Summary

This manuscript investigates the association between over indebtedness and overweight/obesity within a Swiss population, an area notably underexplored in current literature. The authors utilise a cross-sectional design, combining primary data from overindebted individuals seeking advice with secondary data from the Swiss Health Survey, to examine prevalence rates and calculate adjusted odds ratios for overweight and obesity among overindebted persons. The study finds statistically significant, albeit modest, increased risks of overweight and obesity associated with over indebtedness, independent of sex, age, and educational level, and considers potential mediators such as loneliness and sense of control.

Strengths:

- Addresses a significant gap in the literature regarding social determinants of obesity in Switzerland.

- Utilises a novel combination of primary and secondary data sources to enhance the representativeness of overindebted individuals.

- Applies appropriate statistical methods, including multivariate logistic regression, to adjust for confounders.

Limitations:

- Cross-sectional design precludes causal inference.

- Potential misclassification bias due to self-reported measures and the indirect assessment of over indebtedness.

- Limited discussion on the potential reverse causality and residual confounding.

- The sample size of overindebted individuals (n=219) may limit statistical power and generalisability.

---

2. Methodological Critique

Study Design Appropriateness

The cross-sectional observational design is suitable for exploring associations but inherently limited in establishing causality. The authors acknowledge this limitation, which is appropriate. The combination of primary data from overindebted individuals and secondary data from a nationally representative survey is innovative and enhances the study's scope. However, the merging of these datasets raises concerns about comparability and potential selection biases.

Statistical Analysis Methods

The use of odds ratios (ORs) derived from logistic regression models is standard in epidemiological research for binary outcomes such as overweight and obesity. The authors appropriately perform unadjusted and adjusted analyses, controlling for key confounders such as sex, age, and education. The stepwise adjustment approach, including potential mediators like loneliness and sense of control, is methodologically sound.

However, the interpretation of ORs as proxies for relative risk warrants caution, especially given the prevalence of outcomes (~30-46%). When outcomes are common, ORs tend to overestimate the relative risk, which the authors do not explicitly address. Alternative measures such as prevalence ratios or risk ratios (via Poisson regression with robust error variance) could provide more interpretable estimates.

Sample Size and Representativeness

The primary sample of overindebted individuals (n=219) is relatively small, which may limit statistical power and the precision of estimates. The secondary sample from the Swiss Health Survey (n=1,997) is larger and representative, but the merging process and potential differences in data collection methods could introduce bias. The authors recognise the underrepresentation of overindebted individuals in population surveys, which justifies their approach.

Data Collection Methods and Potential Biases

Self-reported height and weight are subject to reporting bias, often leading to underestimation of BMI. The authors mention this but do not quantify its potential impact. The assessment of overindebtedness via a primary survey among debt advice clients is appropriate but may not capture all overindebted individuals, especially those not seeking advice, leading to selection bias.

The potential for misclassification bias exists, particularly if some overindebted individuals are not identified or if general population respondents underreport their debts. The authors suggest that misclassification is likely nondifferential, which would bias results towards the null, implying the true association might be stronger.

3. Results Interpretation

The authors interpret their findings cautiously, noting the statistically significant increased odds of overweight and obesity among overindebted individuals. They correctly highlight that the magnitude of the association is modest and that the cross-sectional nature prevents causal assertions. The discussion on potential confounding factors, such as age and education, and the effects of adjustment, is appropriate.

However, the interpretation could benefit from a more nuanced discussion of the potential reverse causality—whether obesity could contribute to financial hardship—and the implications thereof. The authors briefly mention this but could elaborate further, considering the bidirectional nature of social and health determinants.

The comparison with German data provides valuable context, but the authors should clarify that differences in prevalence rates and effect sizes may also reflect cultural, healthcare, and socioeconomic differences beyond mere sample characteristics.

4. Evaluation of Discussion and Conclusions

The discussion effectively synthesises the findings, acknowledging limitations and situating results within the broader literature. The authors appropriately emphasise the novelty of their work and its public health relevance, particularly the potential role of over indebtedness as a social determinant of health.

The conclusions are generally supported by the data, asserting that over indebtedness is associated with increased risk of overweight and obesity, and suggesting that addressing over indebtedness could have health benefits. The authors rightly caution against overinterpretation due to the cross-sectional design.

Nevertheless, the discussion could be strengthened by explicitly addressing the potential for residual confounding, the limitations of self-reported BMI, and the need for longitudinal studies to establish causality.

5. Recommendations for Improvement

- Methodological Clarity: Clarify the rationale for using ORs as proxies for relative risk, especially given the high prevalence of outcomes, and consider alternative models (e.g., Poisson regression) for more interpretable estimates.

- Address Reverse Causality: Expand discussion on the possibility that obesity may influence financial status, and suggest longitudinal research to disentangle causality.

- Data Quality: Discuss the potential impact of self-reported BMI bias more explicitly, possibly referencing validation studies.

- Sample Representativeness: Elaborate on the potential selection bias introduced by recruiting overindebted individuals from advice centers and how this might influence generalisability.

- Statistical Power: Acknowledge the limited sample size of overindebted individuals and its implications for the robustness of findings.

- Further Analyses: Consider stratified analyses by age and education to explore effect modification.

- Visuals and Data Presentation: Ensure that figures and tables are clearly labelled and accessible, and consider including confidence intervals directly in figures for clarity.

- Discussion Depth: Further explore potential mediators and confounders, and discuss policy implications more explicitly.

6. Contribution to the Field

This study makes a valuable contribution by highlighting a previously underexplored social determinant—over indebtedness—in relation to overweight and obesity within the Swiss context. It broadens the understanding of social inequalities in health and underscores the importance of integrating financial hardship into public health strategies. The methodological approach of combining primary and secondary data sources is innovative and could serve as a model for future research in similar contexts.

7. Overall Recommendation

Major Revisions

While the manuscript addresses an important and novel research question with appropriate methods, significant improvements are necessary to strengthen the validity and clarity of the findings. These include addressing the limitations of the statistical approach, elaborating on potential biases, and providing a more nuanced discussion of causality and policy implications. Upon satisfactory revision, the paper has the potential to make a meaningful contribution to the literature on social determinants of health.

REVIEWER DECLARATION:

This review has been conducted in accordance with standard peer review practices and ethical guidelines. The reviewer declares no conflicts of interest with regard to the research, authors, or subject matter of this manuscript.

Reviewer #2: The study investigates the association between being overindebted and overweight/obese in the kanton of Zürich in Switzerland. It adjusts for the most obvious confounders, age, education and gender. It also investigates two potential mediators, feelings of loneliness and sense of control.

1. It would be good if you could provide an answer to why your research question is interesting and relevant already in the introduction.

2. Relatedly, while the literature on overindebtedness and obesity may be small there is a large literature on poverty and obesity. I would assume that poor people are more often overindebted (even if their debt levels are probably lower than for wealthier people). It seems to me that the association between overindebtedness and obesity should be more relevant if there is an association on top and above that with poverty, in the same way as it is more relevant if there is an association on top and above that with education (which you do show). If not, overindebtedness could be seen as just an alternative measure of poverty.

Ideally you have alternative measures of income or poverty in your data, and you can explore whether a statistically significant association remains when poverty/income is accounted for.

If you do not have comparable measures in both data sources, you could still compare how well overindebtedness predict overweight/obesity compared to how well poverty predicts it in the Swiss Health Survey. You can also compare your estimated association with comparable associations between poverty and overweight/obesity in the literature for Switzerland in particular.

3. It would be good to mention other potential confounders or mediators suggested by the literature, that you cannot investigate. This will clarify the contribution of the paper in relation to the existing literature. Potential confounders or mediators that you cannot include in your study can also be mentioned as a limitation of the study.

4. Sense of control can probably be a confounder just as well as a mediator. It is easy to imagine that a lower sense of control increases both the risk of becoming indebted and of becoming obese. It is also easy to imagine that having become indebted or obese lowers sense of control though. The study does not allow the investigation of directions of causality, but you could be more agnostic about it.

5. It would be good to have more details on your included confounding and mediating variables. It is not clear exactly what variables are used in your regressions presented in Table 2. What does for example Age (1-8) mean? That you include eight different age categories in the regressions? Which categories? Or do you use the categories in Table 1 (only three categories). Why do you use the particular categories that you do? This comment applies also to education, sense of control, and feelings of loneliness.

6. What was the participation rate in the debt advisory center survey? That is, how many participants asked to participate did not do so?

7. Do you have any idea about the likely number of overindebted individuals in the Swiss Health Survey? If the Swiss Health Survey is representative, it should probably be close to population levels? Is there some information in the survey that can be used to proxy the number? Having some idea would be useful to say something about the likely extent of misclassification. Is it likely to be minimal or sizeable?

8. You discuss possible misclassification of exposure – I think that you refer to the possibility of some overindebted people in the Swiss Health Survey here? Clariy this.

9. In your `strengths and weaknesses´ you emphasize that you do not make any causal claims, while you still seem to want to make such claims in your conclusions. You should be more coherent. I think that you should avoid statements that you cannot back with your analysis in the conclusions. You can discuss possible reasons behind the association, but any statement that you cannot back is speculative, which should be clear.

Some small comments:

i) In the introduction: the sentence that the lack of prior studies is “particularly applicable to Switzerland” seems a bit unnecessary if there is only one prior study, from Germany.

ii) The statement about both a systematic underestimation and overestimation is confusing. When you adjust for both education and age the problem disappears, no? It would be more interesting if you could discuss likely confounders that you could not include, and the likely bias on your estimated odds ratio because of that.

iii) I suggest removing the word “finally” in “the study simply wanted to demonstrate and has finally…” . It suggests to the reader that this is something that the literature have long wanted to do, and now finally you have been able to. The word simply also seems a bit unnecessary.

iv) In general think about word choices and language to make it as correct and to the point as possible.

v) Will the data collected at the debt advisory centers be freely available, I see only information about the Swiss Health Survey in the Data Centre.

**********

what does this mean? ). If published, this will include your full peer review and any attached files.

If you choose “no”, your identity will remain anonymous but your review may still be made public.

**Do you want your identity to be public for this peer review?** For information about this choice, including consent withdrawal, please see our Privacy Policy

Reviewer #1: No

Reviewer #2: No

**********

[NOTE: If reviewer comments were submitted as an attachment file, they will be attached to this email and accessible via the submission site. Please log into your account, locate the manuscript record, and check for the action link "View Attachments". If this link does not appear, there are no attachment files.]

While revising your submission, please upload your figure files to the Preflight Analysis and Conversion Engine (PACE) digital diagnostic tool, https://pacev2.apexcovantage.com/ . PACE helps ensure that figures meet PLOS requirements. To use PACE, you must first register as a user. Registration is free. Then, login and navigate to the UPLOAD tab, where you will find detailed instructions on how to use the tool. If you encounter any issues or have any questions when using PACE, please email PLOS at figures@plos.org . Please note that Supporting Information files do not need this step.

Attachment Submitted filename: Referee report oveindebted and obese plos one.pdf

# Author comment: Author response to Decision Letter 1

**DOI:** 10.1371/journal.pone.0342080.r002

13 Jan 2026

Reviewer #1:

Statistical analysis

• “However, the interpretation of ORs as proxies for relative risk warrants caution, especially given the prevalence of outcomes (~30-46%). When outcomes are common, ORs tend to overestimate the relative risk, which the authors do not explicitly address. Alternative measures such as prevalence ratios or risk ratios (via Poisson regression with robust error variance) could provide more interpretable estimates.”

• Recommendation:

“Methodological Clarity: Clarify the rationale for using ORs as proxies for relative risk, especially given the high prevalence of outcomes, and consider alternative models (e.g., Poisson regression) for more interpretable estimates.”

I agree with the reviewer that calculating odds ratios as a proxy for the relative risk of being overweight among overindebted individuals is somewhat limited as overweight is not a rare phenomenon in Switzerland as elsewhere. Odds ratios are good measures for the relative risk when outcomes are rare, but less so when they are common. However, since the only existing and comparable study from another country (Germany) has carried out the same logistic regression analyses (see Discussion section), I would dislike giving it up completely. Nevertheless, I have additionally performed Poisson regression analyses and calculated risk ratios (RRs) as measures of association. The risk ratios as newly and additionally presented estimates in Table 2 show slightly smaller effect sizes than odds ratios. Not surprisingly, this applies mainly for overweight as the much more common outcome.

In the Methods, the Results and the Discussion section different new paragraphs have been formulated and inserted concerning this matter.

Results

• “However, the interpretation could benefit from a more nuanced discussion of the potential reverse causality—whether obesity could contribute to financial hardship—and the implications thereof. The authors briefly mention this but could elaborate further, considering the bidirectional nature of social and health determinants.”

• Recommendation:

“Address Reverse Causality: Expand discussion on the possibility that obesity may influence financial status, and suggest longitudinal research to disentangle causality.”

This has now been more extensively addressed and elaborated in the Discussion section. See a new paragraph with several new references under “Limitations”.

Discussion / Limitations

• “The comparison with German data provides valuable context, but the authors should clarify that differences in prevalence rates and effect sizes may also reflect cultural, healthcare, and socioeconomic differences beyond mere sample characteristics.”

A paragraph on that has been inserted in the Discussion section.

• “Data Quality: Discuss the potential impact of self-reported BMI bias more explicitly, possibly referencing validation studies.”

Done. See a new paragraph on that at the end of the “Limitations” subsection in the Discussion section.

• “Sample Representativeness: Elaborate on the potential selection bias introduced by recruiting overindebted individuals from advice centers and how this might influence generalisability.”

Done (see new paragraph under “Limitations” in the Discussion section).

• “Statistical Power: Acknowledge the limited sample size of overindebted individuals and its implications for the robustness of findings.”

Considering the estimated prevalence of the phenomenon of overindebtedness in Switzerland between 6.5% and 8% (see Hämmig & Herzig 2022) the proportion of overindebted individuals of 10% in the study sample of 2,216 is not too low. The absolute number of 219 overindebted individuals also does not really limit the statistical power or the robustness of the findings. The proportion of overindebted individuals and the findings in the study are comparable with those of the only existing German study on this topic which has a much larger sample size (Münster et al. 2009).

• “Further Analyses: Consider stratified analyses by age and education to explore effect modification.”

The sample size – although not too small – is not large enough to do education- or age-stratified analyses. However, I do not see a theoretical foundation for an effect modification anyway or rather a biological reason for different associations in different age groups or educational levels. And just doing stratified analyses as an explorative approach would be inappropriate anyway in my view.

• “Visuals and Data Presentation: Ensure that figures and tables are clearly labelled and accessible, and consider including confidence intervals directly in figures for clarity.”

Due to small numbers in frequency distributions by age group and educational level confidence intervals are getting partly very large and mostly overlap. Anyway, bar charts in Figures 1 and 2 are not shown to demonstrate that age groups or educational levels differ significantly from one another in this regard but rather to illustrate patterns of distribution and to justify that all relevant study variables (exposure, outcome, mediators/confounders) and studied/considered phenomena (overindebtedness, low sense of control, feelings of loneliness, obesity) go along with each other.

• “Discussion Depth: Further explore potential mediators and confounders, and discuss policy implications more explicitly.”

Done. See different highlighted sentences in the Discussion and the Conclusions section.

Reviewer #2:

• “It would be good if you could provide an answer to why your research question is interesting and relevant already in the introduction.”

Done. See the yellow marked paragraph in the Introduction section.

• “Relatedly, while the literature on overindebtedness and obesity may be small there is a large literature on poverty and obesity. I would assume that poor people are more often overindebted (even if their debt levels are probably lower than for wealthier people). It seems to me that the association between overindebtedness and obesity should be more relevant if there is an association on top and above that with poverty, in the same way as it is more relevant if there is an association on top and above that with education (which you do show). If not, overindebtedness could be seen as just an alternative measure of poverty.

Ideally you have alternative measures of income or poverty in your data, and you can explore whether a statistically significant association remains when poverty/income is accounted for.

If you do not have comparable measures in both data sources, you could still compare how well overindebtedness predict overweight/obesity compared to how well poverty predicts it in the Swiss Health Survey. You can also compare your estimated association with comparable associations between poverty and overweight/obesity in the literature for Switzerland in particular.”

I do not agree. Overindebtedness and poverty in my view are fairly different phenomena, and data used do not include established and common measures of poverty anyway. Overindebtedness is currently increasing in high-income countries and is observed even among better earning individuals and households. Poverty in contrast is excluded by definition among higher earners. Therefore I do not want to make a connection or mixing between the two phenomena and research fields and traditions in the paper.

In my view and in the case and context of Switzerland it’s more a low social or socioeconomic status measured by education and/or income that is associated with overindebtedness and obesity. Income is not available as an identical and comparable measure in both data sets. And education has been considered in the present study.

• “It would be good to mention other potential confounders or mediators suggested by the literature, that you cannot investigate. This will clarify the contribution of the paper in relation to the existing literature. Potential confounders or mediators that you cannot include in your study can also be mentioned as a limitation of the study.”

Since the literature on overindebtedness and obesity is practically inexistent there are no (other) potential confounders or mediators suggested by the literature. Risk or influencing factors of overweight or obesity named in the literature include sex, age, education, income, depression and smoking. I have studied a few (sex, age, education) and considered another two (sense of control, feelings of loneliness) which are not mentioned in the scarce literature but which are found to be related with depression and smoking.

Nevertheless, I have now additionally considered and included depression as another possible mediator or confounder in the regression analyses. Depressive symptoms were assessed in the two merged surveys and data sets. I have not considered depression as another mediator before since it turned out to be strongly associated with one’s sense of control and feelings of loneliness. Apart from depression I don’t see the necessity of introducing or discussing other potential mediators or confounders which I cannot study or for which I don’t have a rationale.

• “Sense of control can probably be a confounder just as well as a mediator. It is easy to imagine that a lower sense of control increases both the risk of becoming indebted and of becoming obese. It is also easy to imagine that having become indebted or obese lowers sense of control though. The study does not allow the investigation of directions of causality, but you could be more agnostic about it.”

I do now consequently talk or write about possible/potential mediators or confounders in connection with the sense of control or feelings of loneliness..

• “It would be good to have more details on your included confounding and mediating variables. It is not clear exactly what variables are used in your regressions presented in Table 2. What does for example Age (1-8) mean? That you include eight different age categories in the regressions? Which categories? Or do you use the categories in Table 1 (only three categories). Why do you use the particular categories that you do? This comment applies also to education, sense of control, and feelings of loneliness.”

For anonymity reasons in the overindebtedness survey age wasn’t asked exactly but rather by age category. In Table 1 these eight categories were reduced to four age groups. The sum score of the Pearlin Mastery Scale ranging from 4 to 16 as used in regression analyses (see Table 2) has been categorized into four degrees of sense of control (from “no” to “high”) in Table 1. And the seven assessed educational levels (see Table 2) were grouped into four categories (see Table 1). See original descriptions and additional clarifications (yellow marked) in “Measures”.

• “What was the participation rate in the debt advisory center survey? That is, how many participants asked to participate did not do so?”

Out of 369 adult clients showing up in the observation period, 219 with sufficient language skills were preselected by the debt advisors. All of the preselected clients who were eligible, basically willing and finally asked to participate in the survey did so (see also Hämmig & Herzig 2022).

• “Do you have any idea about the likely number of overindebted individuals in the Swiss Health Survey? If the Swiss Health Survey is representative, it should probably be close to population levels? Is there some information in the survey that can be used to proxy the number? Having some idea would be useful to say something about the likely extent of misclassification. Is it likely to be minimal or sizeable?”

No, there is absolutely no information that could be used to proxy the number of overindebted individuals in the Swiss Health Survey. We just know from (other) population surveys and socially disadvantaged groups that marginalized and stigmatized people like unemployed, working poor or overindebted individuals usually are strongly underrepresented in population-based surveys. Therefore the extent of misclassification is presumably minimal rather than sizeable.

• “You discuss possible misclassification of exposure – I think that you refer to the possibility of some overindebted people in the Swiss Health Survey here? Clariy this.”

Yes, because there must be some overindebted people in a random sample of the general population which cannot be identified and classified as such, misclassification is likely to have occurred.

• “In your `strengths and weaknesses´ you emphasize that you do not make any causal claims, while you still seem to want to make such claims in your conclusions. You should be more coherent. I think that you should avoid statements that you cannot back with your analysis in the conclusions. You can discuss possible reasons behind the association, but any statement that you cannot back is speculative, which should be clear.”

I have shortened and reformulated the according paragraph in the “Conclusion” subsection.

Some small comments:

i) In the introduction: the sentence that the lack of prior studies is “particularly applicable to Switzerland” seems a bit unnecessary if there is only one prior study, from Germany.

The according sentence is now deleted.

ii) The statement about both a systematic underestimation and overestimation is confusing. When you adjust for both education and age the problem disappears, no? It would be more interesting if you could discuss likely confounders that you could not include, and the likely bias on your estimated odds ratio because of that.

I don’t understand this point. Systematically underestimated effects due to misclassification of exposure (overindebtedness) do not disappear by adjusting for age or education. They have to be considered and acknowledged.

iii) I suggest removing the word “finally” in “the study simply wanted to demonstrate and has finally…” . It suggests to the reader that this is something that the literature have long wanted to do, and now finally you have been able to. The word simply also seems a bit unnecessary.

I agree and have deleted the word.

iv) In general think about word choices and language to make it as correct and to the point as possible.

I tried to do so and to do better in this regard when revising the manuscript.

v) Will the data collected at the debt advisory centers be freely available, I see only information about the Swiss Health Survey in the Data Centre.

No, in contrast to the data of the Swiss Health Survey the data collected from the debt advisory centers are not freely available.

Attachment Submitted filename: renamed_ae1c6.pdf

# Editor report: Decision Letter 1

**DOI:** 10.1371/journal.pone.0342080.r003

**Authors:** Fabrizio Ferretti

19 Jan 2026

Being overindebted and overweight in Switzerland – A largely unexplored association in an understudied population

PONE-D-25-13328R1

Dear Dr. Hämmig,

We’re pleased to inform you that your manuscript has been judged scientifically suitable for publication and will be formally accepted for publication once it meets all outstanding technical requirements.

Within one week, you’ll receive an e-mail detailing the required amendments. When these have been addressed, you’ll receive a formal acceptance letter and your manuscript will be scheduled for publication.

An invoice will be generated when your article is formally accepted. Please note, if your institution has a publishing partnership with PLOS and your article meets the relevant criteria, all or part of your publication costs will be covered. Please make sure your user information is up-to-date by logging into Editorial Manager at Editorial Manager®  and clicking the ‘Update My Information' link at the top of the page. For questions related to billing, please contact billing support .

If your institution or institutions have a press office, please notify them about your upcoming paper to help maximize its impact. If they’ll be preparing press materials, please inform our press team as soon as possible -- no later than 48 hours after receiving the formal acceptance. Your manuscript will remain under strict press embargo until 2 pm Eastern Time on the date of publication. For more information, please contact onepress@plos.org.

Kind regards,

Fabrizio Ferretti, Ph.D.

Academic Editor

PLOS One

Additional Editor Comments (optional):

Reviewers' comments:

# Editor report: Acceptance letter

**DOI:** 10.1371/journal.pone.0342080.r004

**Authors:** Fabrizio Ferretti

PONE-D-25-13328R1

PLOS One

Dear Dr. Hämmig,

I'm pleased to inform you that your manuscript has been deemed suitable for publication in PLOS One. Congratulations! Your manuscript is now being handed over to our production team.

At this stage, our production department will prepare your paper for publication. This includes ensuring the following:

* All references, tables, and figures are properly cited

* All relevant supporting information is included in the manuscript submission,

* There are no issues that prevent the paper from being properly typeset

You will receive further instructions from the production team, including instructions on how to review your proof when it is ready. Please keep in mind that we are working through a large volume of accepted articles, so please give us a few days to review your paper and let you know the next and final steps.

Lastly, if your institution or institutions have a press office, please let them know about your upcoming paper now to help maximize its impact. If they'll be preparing press materials, please inform our press team within the next 48 hours. Your manuscript will remain under strict press embargo until 2 pm Eastern Time on the date of publication. For more information, please contact onepress@plos.org.

You will receive an invoice from PLOS for your publication fee after your manuscript has reached the completed accept phase. If you receive an email requesting payment before acceptance or for any other service, this may be a phishing scheme. Learn how to identify phishing emails and protect your accounts at https://explore.plos.org/phishing.

If we can help with anything else, please email us at customercare@plos.org.

Thank you for submitting your work to PLOS ONE and supporting open access.

Kind regards,

PLOS ONE Editorial Office Staff

on behalf of

Dr. Fabrizio Ferretti

Academic Editor

PLOS One
